# Priming stem cells with protein kinase C activator enhances early stem cell-chondrocyte interaction by increasing adhesion molecules

**DOI:** 10.1186/s40659-018-0191-6

**Published:** 2018-11-01

**Authors:** Dong-Sik Chae, Chang Youn Lee, Jiyun Lee, Hyang-Hee Seo, Chong-Hyuk Choi, Seahyoung Lee, Ki-Chul Hwang

**Affiliations:** 1 Department of Medicine, The Graduate School, Yonsei University, Seoul, South Korea; 2 Department of Orthopedic Surgery, International St. Mary’s Hospital, Catholic Kwandong University College of Medicine, Incheon, South Korea; 3 Department of Integrated Omics for Biomedical Sciences, Yonsei University, Seoul, South Korea; 4 Brain Korea 21 PLUS Project for Medical Science, Yonsei University, Seoul, South Korea; 5 Department of Orthopedic Surgery, Yonsei University College of Medicine, 50, Yonsei-ro, Seodaemun-gu, Seoul, 03722 Republic of Korea; 6 Institute for Bio-Medical Convergence, College of Medicine, Catholic Kwandong University, Gangneung-si, Gangwon-do South Korea

**Keywords:** ADSC, PMA, Cellular adhesion, Chondrocyte, Osteoarthritis

## Abstract

**Background:**

Osteoarthritis (OA) can be defined as degradation of articular cartilage of the joint, and is the most common degenerative disease. To regenerate the damaged cartilage, different experimental approaches including stem cell therapy have been tried. One of the major limitations of stem cell therapy is the poor post-transplantation survival of the stem cells. Anoikis, where insufficient matrix support and adhesion to extracellular matrix causes apoptotic cell death, is one of the main causes of the low post-transplantation survival rate of stem cells. Therefore, enhancing the initial interaction of the transplanted stem cells with chondrocytes could improve the therapeutic efficacy of stem cell therapy for OA. Previously, protein kinase C activator phorbol 12-myristate 13-acetate (PMA)-induced increase of mesenchymal stem cell adhesion via activation of focal adhesion kinase (FAK) has been reported. In the present study, we examine the effect PMA on the adipose-derived stem cells (ADSCs) adhesion and spreading to culture substrates, and further on the initial interaction between ADSC and chondrocytes.

**Results:**

PMA treatment increased the initial adhesion of ADSC to culture substrate and cellular spreading with increased expression of adhesion molecules, such as FAK, vinculin, talin, and paxillin, at both RNA and protein level. Priming of ADSC with PMA increased the number of ADSCs attached to confluent layer of cultured chondrocytes compared to that of untreated ADSCs at early time point (4 h after seeding).

**Conclusion:**

Taken together, the results of this study suggest that priming ADSCs with PMA can increase the initial interaction with chondrocytes, and this proof of concept can be used to develop a non-invasive therapeutic approach for treating OA. It may also accelerate the regeneration process so that it can relieve the accompanied pain faster in OA patients. Further in vivo studies examining the therapeutic effect of PMA pretreatment of ADSCs for articular cartilage damage are required.

## Background

Osteoarthritis (OA) is the most common degenerative disease and can be defined as degradation of articular cartilage of the joint [1]. Treatment for OA generally uses non-steroidal anti-inflammatory drugs (NSAIDs) and hyaluronic acid injections, but these options are mainly to relieve pain rather than regenerate the damaged cartilage [2]. Consequently, additional approaches such as stem cell therapy have been tested for the regeneration of injured articular cartilage [3–5]. Among different types of stem cells, adipose-derived stem cells (ADSCs) are considered as good substitute for bone marrow mesenchymal stem cells (MSCs) due to easy isolation protocol, yet characteristics similar to those of MSCs such as multi-lineage cell differentiation capacity and regenerative potential [6]. However, one of the major limitations of stem cell therapy in regenerative medicine in general is the poor survival rate of the stem cells transplanted into damaged tissues, which decreases the therapeutic efficacy of the transplanted stem cells [7, 8].

The low survival rate of the transplanted stem cells in vivo has been linked to anoikis where insufficient matrix support and adhesion to extracellular matrix (ECM) causes apoptotic cell death [9]. To improve cell adhesion, number of approaches had been tried including the enhancement of anti-apoptotic signals [10–13], modulation of cell adhesion molecules [14–16], hypoxic preconditioning [17–19], and pretreatment of various growth factors, cytokines, and chemicals [20–23]. A previous study demonstrated that the protein kinase C (PKC) activator phorbol 12-myristate 13-acetate (PMA) increased cell adhesion of rat MSCs via activation of focal adhesion kinase (FAK) and the PMA-pretreated rat MSCs improved cardiac function following myocardial infarction [24].

The activation of PKC plays important roles in cytoskeletal rearrangement, cell proliferation, survival, cell death [25], and the activation of FAK [26], which results in the modulation of cell adhesion and motility by recruiting other focal adhesion proteins including paxillin [27, 28]. Therefore, priming stem cells with PMA may enhance the initial interaction between transplanted stem cells and host tissue cells, further increasing the therapeutic efficacy. In the present study, we examined the effect of PMA pretreatment on ADSC adhesion and spreading and further investigated the effect of priming ADSCs with PMA on the initial interaction between ADSCs and chondrocyte.

## Methods

### Culture of human adipose-derived stem cells (ADSCs)

Human adipose-derived stem cells (hADSCs) were purchased from Invitrogen (Waltham, MA, USA). hADSCs were cultured according to the manufacturer’s instructions. High glucose-Dulbecco’s modified Eagle’s medium (DMEM; Gibco, Waltham, MA, USA) containing 10% fetal bovine serum (FBS; Gibco) and 1% antibiotics (Gibco) were used to culture and maintain the cells. The media were changed every 3 days, and the cells were passaged using 0.25% trypsin (Gibco) when they reached 80–90% confluency. The cells from passages 6–10 were used for experiments.

### Culture of human chondrocytes

Human chondrocytes were purchased from PromoCell (Heidelberg, Germany). The cells were cultured according to the manufacturer’s instructions. Chondrocyte growth medium (c-27101, PromoCell) was used to culture and maintain the cells. The media were changed every 3 days, and the cells were passaged using 0.25% trypsin (Gibco) when they reached 80–90% confluency. The cells from passages up to 6 were used for experiments.

### PMA treatment

We examined the effect of PMA pretreatment on ADSC adhesion and spreading and further investigated the effect of priming ADSCs with PMA on the initial interaction between ADSCs and chondrocyte. To activate PKC in ADSCs, we used a well-known PKC activator PMA (Sigma-Aldrich, St. Louis, MO). PMA was dissolved in dimethyl sulfoxide (DMSO) at a concentration of 100 μM (stock solution). For cell treatment, 100 μM stock solution was diluted in DMEM to the desired concentration.

### Cell viability assay

To measure cell viability, 5 × 10^3^ hADSCs cells were plated in a 96-well plate. The cells were treated with varying concentrations of PMA (10, 20, 50, and 100 nM) PMA and incubated for 24 h. Highly water-soluble tetrazolium salt WST-8 (CCK-8, Dogen, Seoul, Korea) was added to each well to a final concentration of 0.5 mg/mL. WST-8 is reduced by dehydrogenases in cells to give an orange colored formazan, and the amount of the formazan dye generated by dehydrogenases in cells is directly proportional to the number of living cells. The WST-8-treated cells were incubated for additional 2 h. The amount of formazan formed was measured using a microplate reader at 450 nm (Thermo Fisher Scientific Waltham, MA, USA).

### Evaluation of cell adhesion

To evaluate ADSC adhesion, after 4 h of pretreatment increasing concentrations of PMA (or DMSO for vehicle group) as a suspension in culture medium, the cells were centrifuged and washed with PBS. The cells were seeded in a 6 well plate (5 × 10^4^ cells/well) without any further PMA treatment. Four 4 h after the seeding, the attached cells were carefully washed with PBS three times, and then four separate fields were photographed with a phase contrast microscope for counting.

### Evaluation of cell spreading

For spreading assays, ADSCs were pretreated with PMA (or DMSO for vehicle group) as a suspension in culture medium for 4 h in a cell culture incubator. The PMA pretreated ADSCs were seeded in a 4 well chamber slide at a density of 1 × 10^3^ cells/well and cultured for 6 h. To determine the extent of ADSC spreading, the cells were washed 3 times with PBS, fixed with 3% formaldehyde, stained with Coomassie blue [0.1% solution of Coomassie brilliant blue R250 in 40% methanol: acetic acid: water, 46.5:7:46.5 (v/v/v)], and rinsed with distilled water [29]. Finally, the four separate fields were photographed using a phase contrast microscope.

### F-actin staining

In order to visualize ADSC’s cytoskeleton, the 4 h PMA (or DMSO for vehicle group) pretreated ADSCs were seeded in a 4 well chamber slide at a density of 1 × 10^3^ cells/well and cultured for 6 h. The cells were washed with PBS and fixed in 10% formalin solution for 10 min at room temperature. After PBS wash, the cells were permeabilized in 0.1% Triton X-100 for 5 min and then PBS washed. After 30 min of blocking in 1% bovine serum albumin in PBS, and F-actin was stained with Texas Red-X phalloidin (Invitrogen, Waltham, MA, USA) overnight at 4 °C. Immunofluorescence was detected by confocal microscopy (LSM710; Carl Zeiss Microscopy GmbH, Jena, Germany).

### Wound healing assay (migration assay)

ADSCs were plated at a density of 8 × 10^4^ cells/well in six-well plates. After the cells had reached 70% confluence, wounds were produced by scratching with 200 μL pipette tips. The medium was replaced with or without PMA (100 nM), and the cells were incubated for up to 24 h (1 day). Images were captured using an Axiovert 40 °C inverted microscope (Carl Zeiss, Germany) equipped with a Powershot A640 digital camera (Canon, Japan) at 4 h and 24 h.

### Seeding ADSCs on top of cultured chondrocytes

Chondrocytes were cultured in 6 well plates until they became confluent. Meantime, cultured ADSCs were trypsinized and the cells were suspended in 3 aliquots; (1) untreated ADSC, (2) PMA + ADSCs, and (3) PMA pretreated ADSCs (2 × 10^3^ cells/mL each). Only the PMA pretreated ADSC group was incubated with 100 nM of PMA for 4 h. Meanwhile, the untreated ADSCs and PMA + ADSCs were kept in an incubator without PMA for the 4 h. After 4 h, Hoechst dye (2 μg/mL) was added to all groups and the cell suspensions were kept in an incubator for 30 min. The cell suspensions were centrifuged and the cell pellets were washed with PBS 3 times. For the PMA + ADSC group, 100 nM of PMA was added to the cell suspension just prior to the seeding. The Hoechst-stained ADSCs (2 × 10^3^ cells per well) were seeded on top of confluent chondrocytes and cultured for additional 4 h. Unattached cells were washed 3 times with fresh medium, and Hoechst-stained ADSCs were imaged under fluorescent microscopy.

### Reverse transcription polymerase chain reaction (RT-PCR)

Briefly, the cells were seeded to a well of 6-well plate in DMEM containing 100 nM PMA and incubated in a humidified atmosphere of 95% air and 5% CO_2_ (Forma Scientific, USA) at 37 °C. The cells were collected at 4 h after the initial seeding for reverse transcriptase PCR. Total RNA was extracted using TRIzol reagent (Ambion, Waltham, MA, USA) according to the manufacturer’s instructions. Complementary DNA (cDNA) was synthesized from RNA by AMV reverse transcriptase in provided in a RT system kit (Promega, Fitchburg, WI, USA). The primer sequences used were as follows: human PTK2 (FAK, NCBI reference sequence: NM_001199649.1), forward: 5′-TTA TTG GCC ACT GTG GAT GA-3′, reverse: 5′-TAC TCT TGC TGG AGG CTG GT-3′; human TLN1 (Talin1, NCBI reference sequence: NM_006289.3), forward: 5′-TCT CCC AAA ATG CCA AGA AC-3′, reverse: 5′-CTC CAC TAG CCC TTG CTG TC-3′; human VCL (vinculin, NCBI reference sequence: NM_003373.3), forward: 5′-GCC AAG CAG TGC ACA GAT AA-3′, reverse: 5′-AGG TTC TGG GCA TTG TGA AC-3′, human PXN (paxillin, NCBI reference sequence: NM_001080855.2), forward: 5′-GGA GTC TAC CAC CTC CCA CA-3′, reverse: 5′-CCA CTG GTC TAA GGG GTC AA-3′, human GAPDH (NCBI reference sequence: NM_001256799.2), forward: 5′-CAT GGG TGT GAA CCA TGA GAA-3′, reverse: 5′-GGT CAT GAG TCC TTC CAC GAT-3′.

### Western blot

For western blot, the cells were collected at 12 h after the initial seeding with or without PMA in the culture medium. Cells were washed once in PBS and lysed for 20 min in a lysis buffer (cell signaling) containing 20 mM Tris (pH 7.5), 150 mM NaCl, 1 mM Na_2_EDTA, 1 mM EGTA, 1% Triton, 2.5 mM sodium pyrophosphate, 1 mM β-glycerophosphate, 1 mM Na_3_VO_4_, 1 mg/mL leupeptin, and 1 mM PMSF. The cell lysate was centrifuged at 12,000*g* for 10 min to obtain a supernatant. The protein concentration was measured using a Bradford protein assay kit (BioRad). The membrane was blocked with Tris-buffered saline-tween 20 (TBS-T, 0.1% Tween 20) containing 5% fat-free powdered milk for 1 h at room temperature and then washed twice with TBS-T. Next, the membrane was incubated overnight at 4 °C with primary antibodies against pFAK, FAK, and vinculin (1:1000 dilution, Santa Cruz Biotechnology, Inc.), paxillin (1:500 dilution, Millipore), talin (1:500 dilution, Abcam, Cambridge, MA), and β-actin (1:10,000 dilution, Santa Cruz Biotechnology, Inc.). The membrane was washed 3 times with TBS-T for 10 min each and then incubated with secondary antibodies for 1 h at room temperature. The used secondary antibodies were mouse anti-goat-HRP (1:5000 dilution), goat anti-mouse-HRP (1:4000 dilution), and goat anti-rabbit-HRP (1:2000 dilution, Enzo Life Sciences, Farmingdale, NY). After thorough washing, a band was detected using enhanced chemiluminogenic (ECL) reagent (GE Healthcare Life Sciences). The intensity of the band was quantified using ImageJ 1.40g software (NIH).

### Statistical analysis

Quantitative data were expressed as the mean ± S.E.M. For statistical analysis, Student’s t-test was used for 2 group comparison and one-way ANOVA with Bonferroni correction was performed using OriginPro 8 SR4 software (ver. 8.0951, OriginLab Corporation, USA) if there were more than 3 groups. A *p* value of < 0.05 was considered statistically significant.

## Results

### Effect of PMA on the viability of ADSCs

**Fig. 1:**
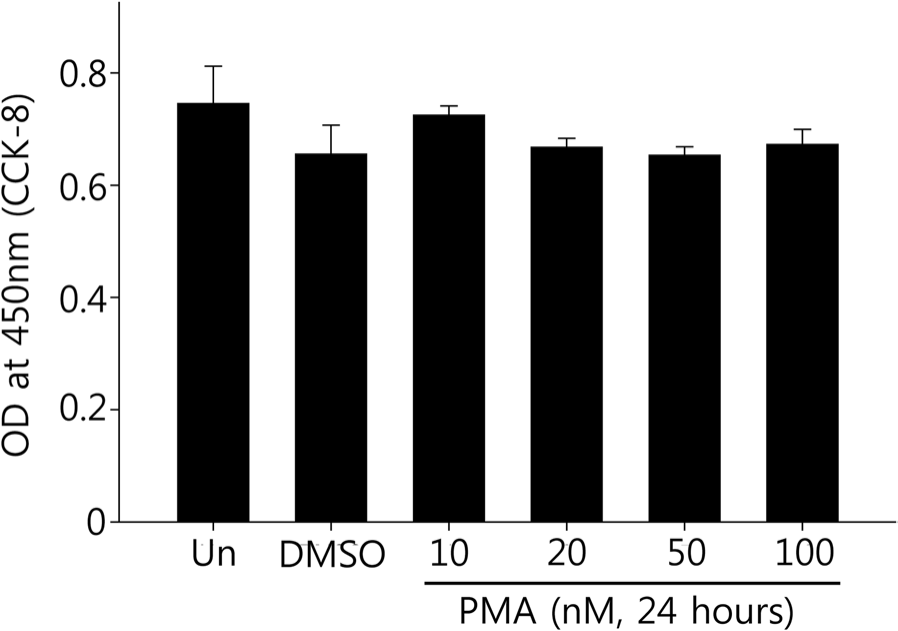
The effect of varying concentrations of PMA on the viability of ADSCs. To test whether PMA itself has any cytotoxic effect on ADSCs, the cells were cultured in a 96 well plate (5 × 10^3^ cells/well) and treated with either vehicle (0.1% DMSO) or varying concentrations of PMA as indicated for 24 h. Cell viability was measured by using CCK-8 kit. The quantitative data were expressed as the mean ± S.E.M of at least 3 independent experiments. *Un* untreated control

PMA cytotoxicity on ADSCs was assayed by treating with increasing concentrations of PMA (10, 20, 50, and 100 nM) over 24 h and determining cell viability using CCK-8 kit. As can be observed in Fig. 1, vehicle (0.1% DMSO) and PMA treatments did not induce statistically significant reductions of cell viability in the concentration range tested (Fig. 1).

### Effect of PMA on the adhesion of ADSC to culture substrate

**Fig. 2:**
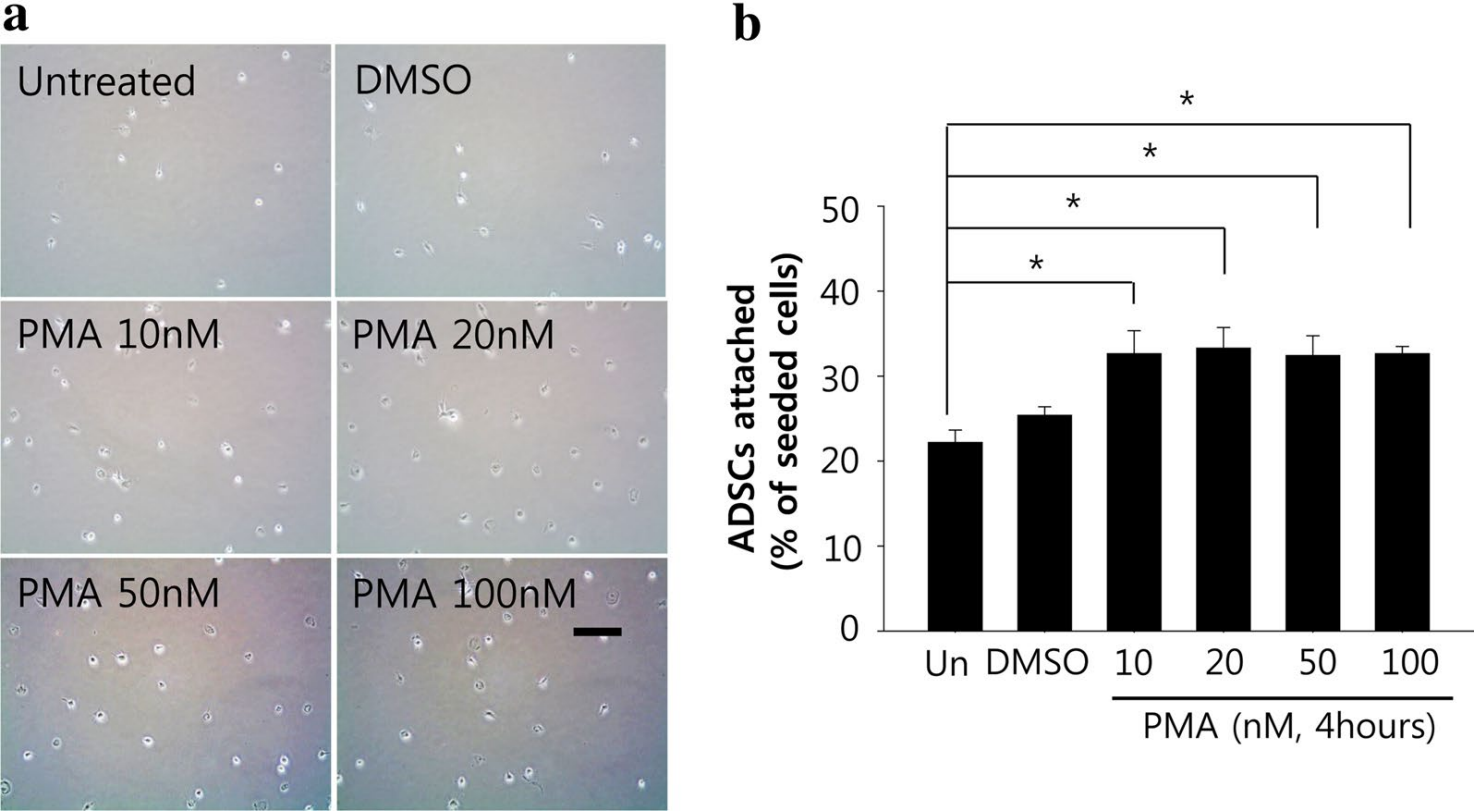
PMA pretreatment increases initial attachment of ADSCs to culture substrate. **a** Representative images of ADSCs attached to culture substrate with or without 4 h of PMA pretreatment. Scale bar = 200 μm. **b** Number of ADSCs attached to culture substrate was counted (per field). The quantitative data were expressed as the mean ± S.E.M of at least 3 independent experiments. *p < 0.05

To examine the effect of PMA on ADSC adhesion to culture substrate, cells were treated with varying concentrations of PMA in suspension for 4 h, and seeded in a 6 well plate (5 × 10^4^ cells/well). The cells were allowed to attach to the culture plate for 4 h and the images of cells were taken for counting (Fig. 2a). According to the data, PMA treatment significantly increased the number of attached ADSCs (32.64 ± 2.10% of initially seeded cells) compared to both untreated (22.18 ± 3.59%) and vehicle (25.38 ± 2.48%) treated cells. However, there was no statistically significant dose-dependent effect among groups treated with different concentrations of PMA (Fig. 2b). Since the 100 nM group showed no significant cytotoxicity and had the smallest intra-sample variation, 100 nM of PMA was used for further experiments.

### Effect of PMA pretreatment on the spreading of ADSCs

**Fig. 3:**
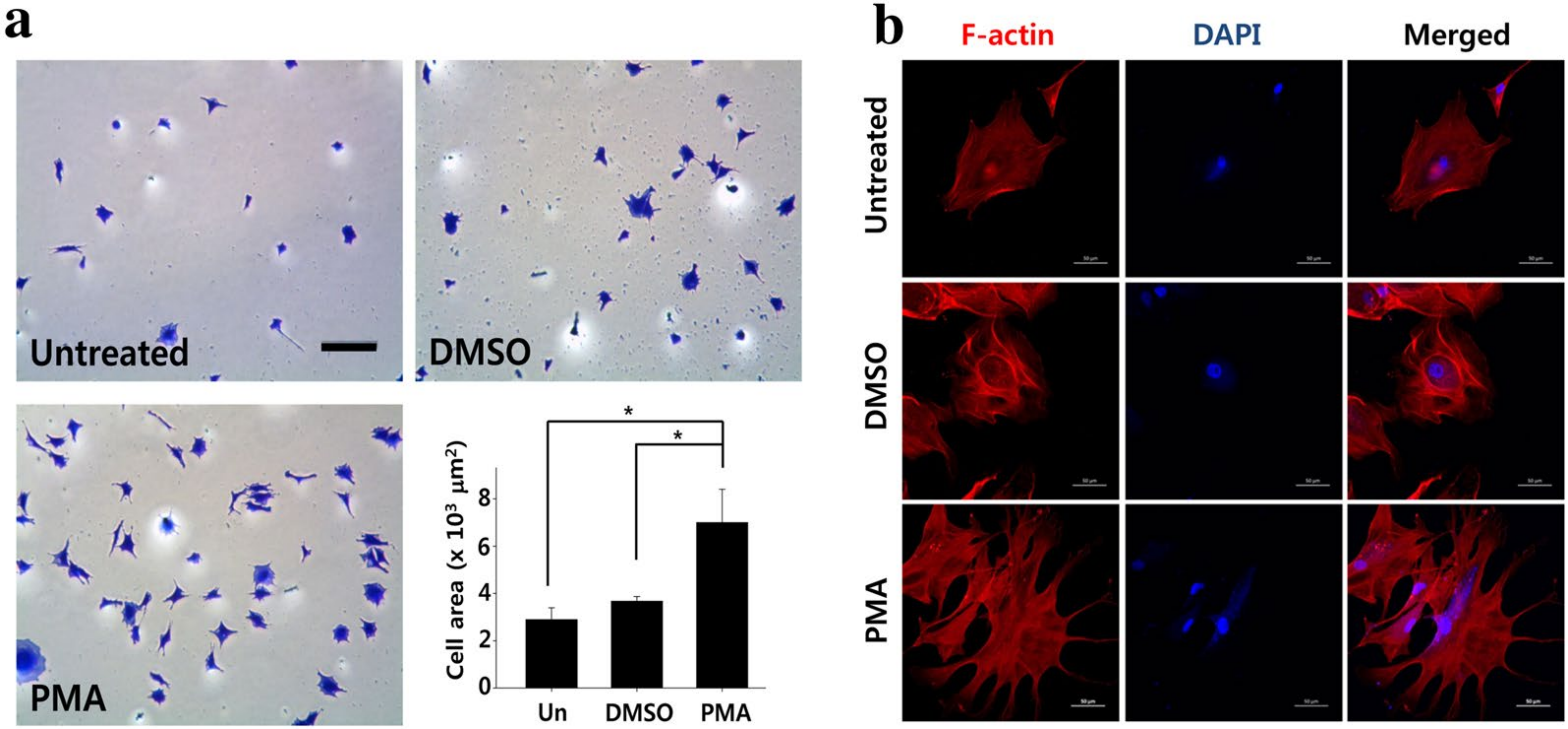
PMA pretreatment enhances cellular spreading of ADSCs. ADSCs pretreated with PMA (100 nM) for 4 h, and then the cells were seeded in a 4 well chamber slide (10^3^ cells/well) and allowed to attach and spread for 6 h. **a** Attached cells were fixed and Coomassie blue stained for clear visualization. After taking images of stained cells, the areas of individual cells were measured. The quantitative data were expressed as the mean ± S.E.M of at least 3 independent experiments. Scale bar = 200 μm, *p < 0.05. **b** F-actin of attached cells was stained with Texas Red-X phalloidin. Scale bar = 50 μm

To further examine the effect of PMA on ADSC adhesion, the PMA pretreated ADSCs (100 nM, 4 h) were seeded and allowed to attach and spread for 6 h, fixed, and stained with Coomassie blue for clear visualization. Compared to both untreated controls and vehicle treated group, PMA (100 nM) treated group showed increased number of cells attached, and more spread (blunt round vs. sharp pointy extensions) morphology (Fig. 3a). Furthermore, the cell area (Coomassie blue stained area) significantly increased with PMA treatment compared to that of both untreated control and vehicle treated group (2.89 ± 1.23 vs. 3.67 ± 0.50 vs. 6.90 ± 3.54 × 10^3^ μm^2^, untreated, vehicle treated, and PMA treated, respectively). To better visualize the cytoskeleton of ADSCs, F-actin staining was performed, and the results showed that PMA pretreated increased filopodia formation (Fig. 3b).

### PMA has no significant effect on cellular migration of ADSCs

**Fig. 4:**
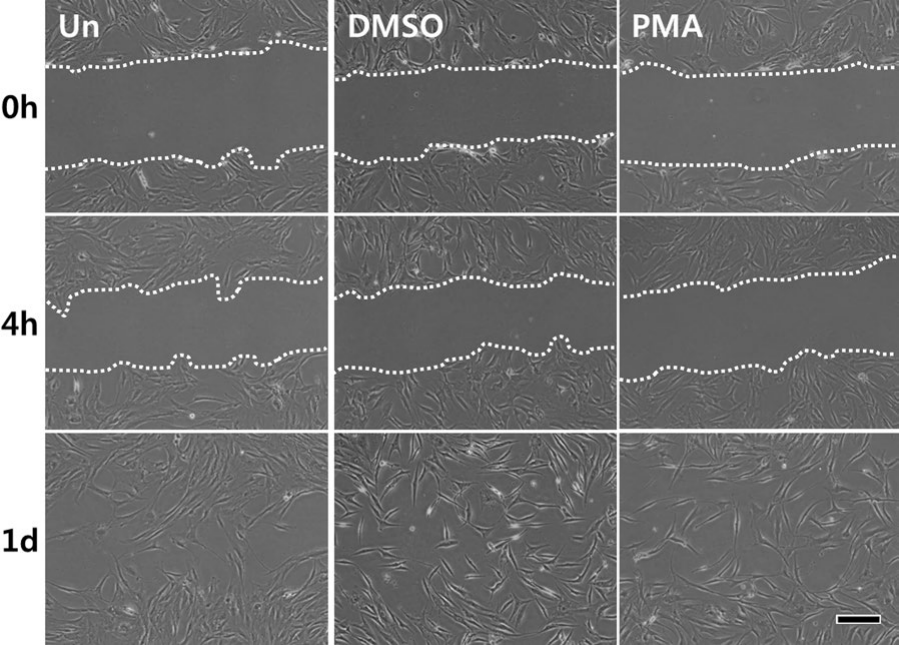
Effect of PMA on the migration of ADSCs. After ADSCs reached approximately 70% confluence, wounds were created using a yellow pipette tip. The cells were cultured with or without PMA (100 nM) for up to 24 h and the images were taken at 4 and 24 h after the wounds were created. Scale bar = 200 μm. White dotted line shows the migrating edge

To examine whether PMA had any effect on ADSC migration, in vitro wound healing assay was conducted. When the cells reached approximately 70% confluence, wounds were created using a 200 μL pipette tip and the cells were allowed to migrate for additional 24 h with or without PMA (100 nM) in the culture media. As shown in the Fig. 4, there was no prominent effect of PMA on the migration of ADSCs observed, regardless of the time after the wound formation (Fig. 4).

### PMA pretreatment enhances the initial interaction between ADSCs and chondrocytes

**Fig. 5:**
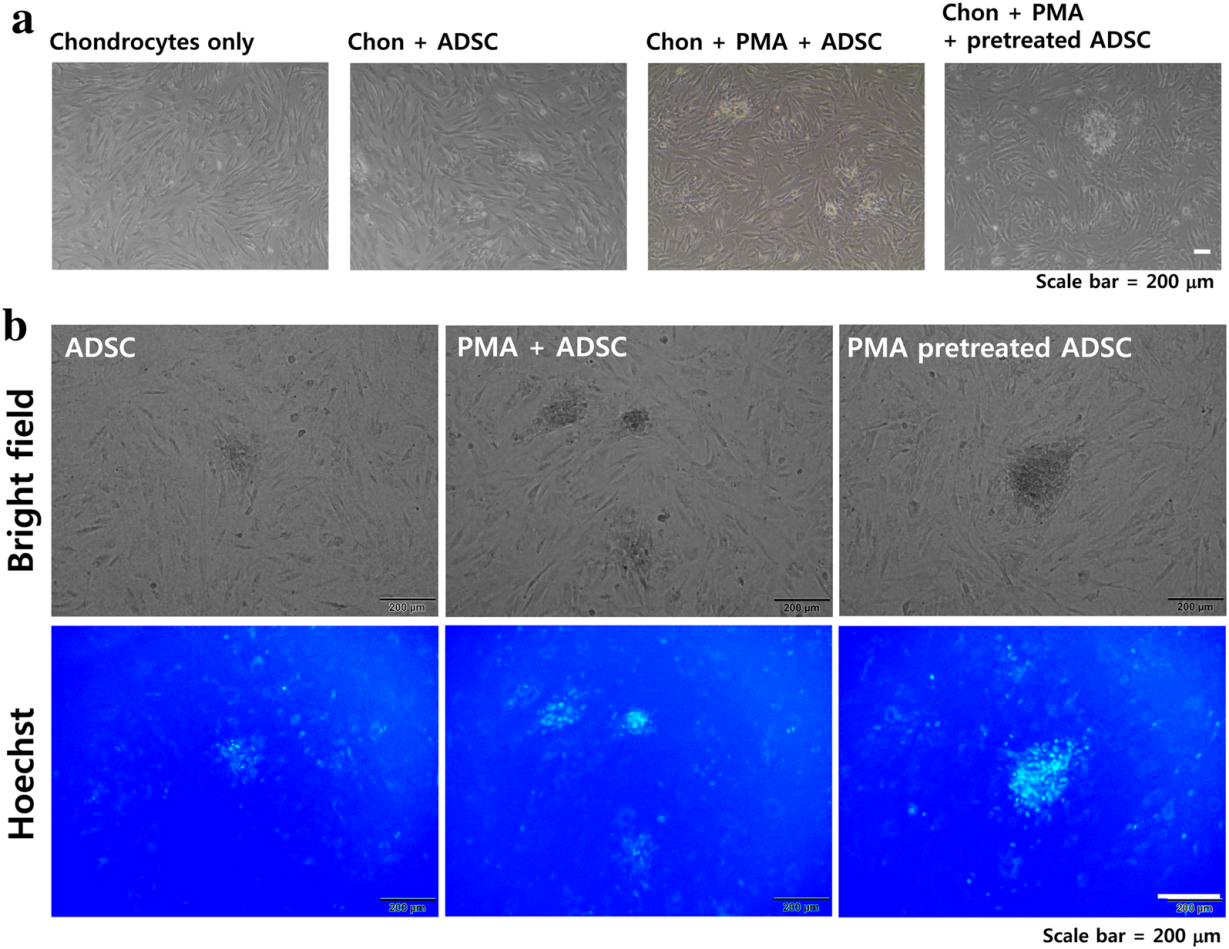
The effect of PMA treatment on the interaction between ADSCs and chondrocytes. ADSCs with or without PMA treatment were seeded on top of the layer of confluent chondrocytes and incubated 4 h after the seeding. **a** After unattached cells were washed with PBS, images were taken using an optical microscope. **b** Hoechst-stained ADSCs were visualized under fluorescent microscopy. ADSC: untreated ADSC, ADSC + PMA: PMA (100 nM) added upon seeding, PMA pretreated ADSC: ADSCs pretreated with PMA (100 nM, 4 h) prior to seeding. To label ADSCs, ADSCs were stained with Hoechst dye (2 μg/mL) for 30 min followed by 3 washes with PBS

To examine whether PMA treatment could enhance the initial cell–cell interaction between ADSCs and chondrocytes, Hoechst-labeled ADSCs with different PMA treatments (4 h of incubation with PMA prior to seeding and PMA treatment upon seeding) were seeded on top of confluent layer of chondrocyte. According to the results, ADSCs pretreated with PMA for 4 h had a tendency to clump together compared to the untreated ADSCs as shown in Fig. 5a. Although ADSCs treated with PMA upon seeding also showed formation of clumps, it was smaller than ones formed in the PMA pretreated ADSCs. Further examination using fluorescent microscopy indicated that the number of ADSCs attached was increased by both PMA treatments (4 h of incubation with PMA prior to seeding and PMA treatment upon seeding) but the location of Hoechst-positive ADSCs was not evenly distributed because they formed clumps (Fig. 5b). Regarding the effect of PMA on chondrocytes, there are only a handful of previous studies directly or indirectly examined the effect of PMA (or PKC activation) on chondrocytes. A relatively recent review indicated that a chronic PMA treatment negatively affects chondrogenesis by downregulating PKC expression [30]. However, since our experimental design involves 4 h of PMA treatment, no significant morphological changes or cell death was observed.

### PMA increases the expression of cell adhesion molecules in ADSCs

**Fig. 6:**
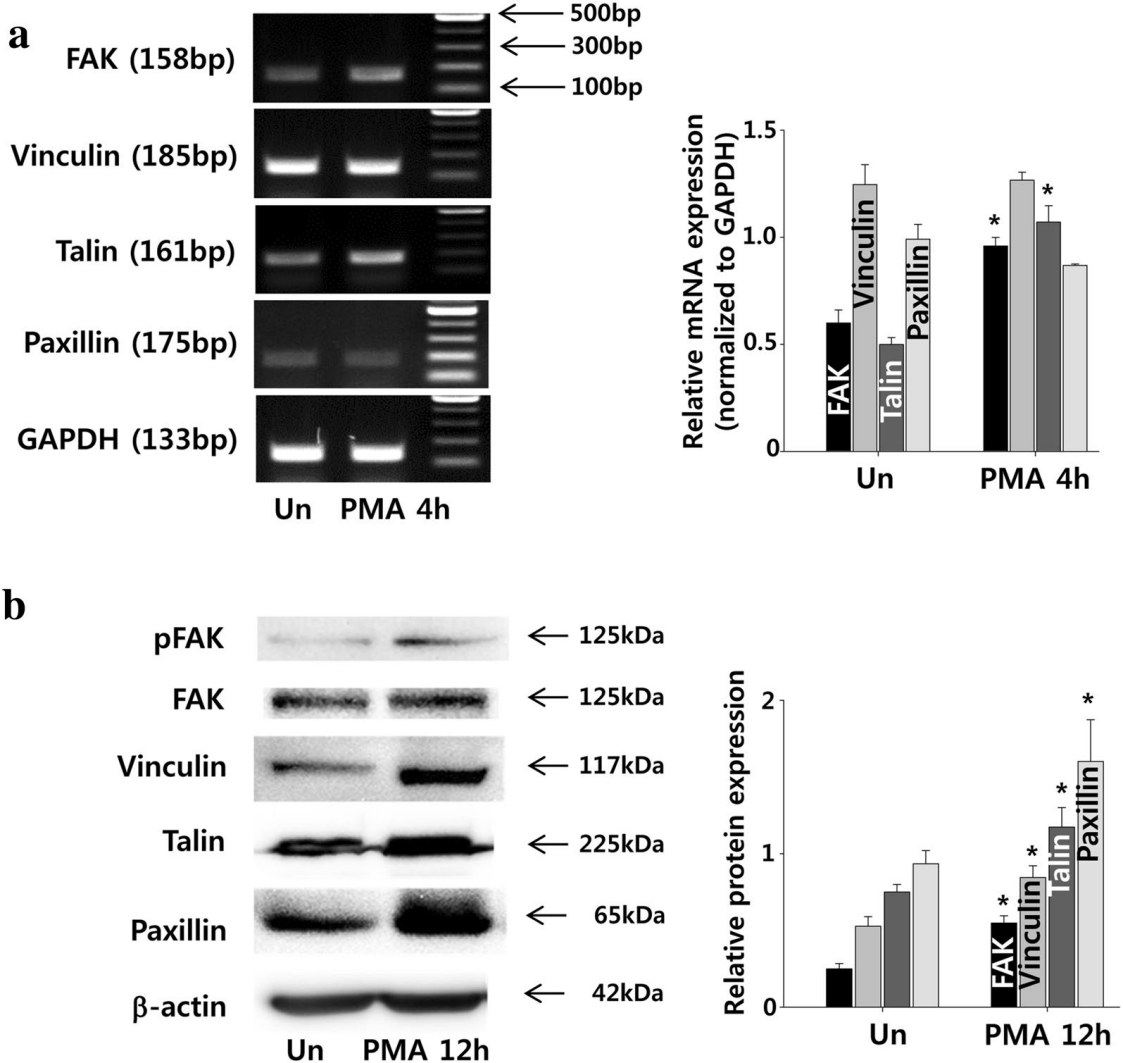
Effect of PMA on the expressions of adhesion molecules in ADCS. ADSCs were treated with PMA (100 nM) up to 12 h. **a** For RT-PCR, samples were collected at 4 h. The mRNA expression of adhesion molecules were normalized to GAPDH. **b** For western blot analysis, samples were collected at 12 h. The expression of phosphorylated FAK (pFAK) was normalized to FAK and expressions of other molecules were normalized to β-actin. The quantitative data were expressed as the mean ± S.E.M of at least 3 independent experiments. *p < 0.05 compared to untreated control

Examination of adhesion-related gene expression using RT-PCR indicated that the expressions of FAK and talin significantly increased with 4 h of PMA treatment, while the expressions of vinculin and paxillin remained unaffected (Fig. 6a). On the other hand, protein expressions of vinculin and paxillin increased at 12 h (Fig. 6b).

### PMA increases the expression of anoikis resistant molecules in ADSCs

**Fig. 7:**
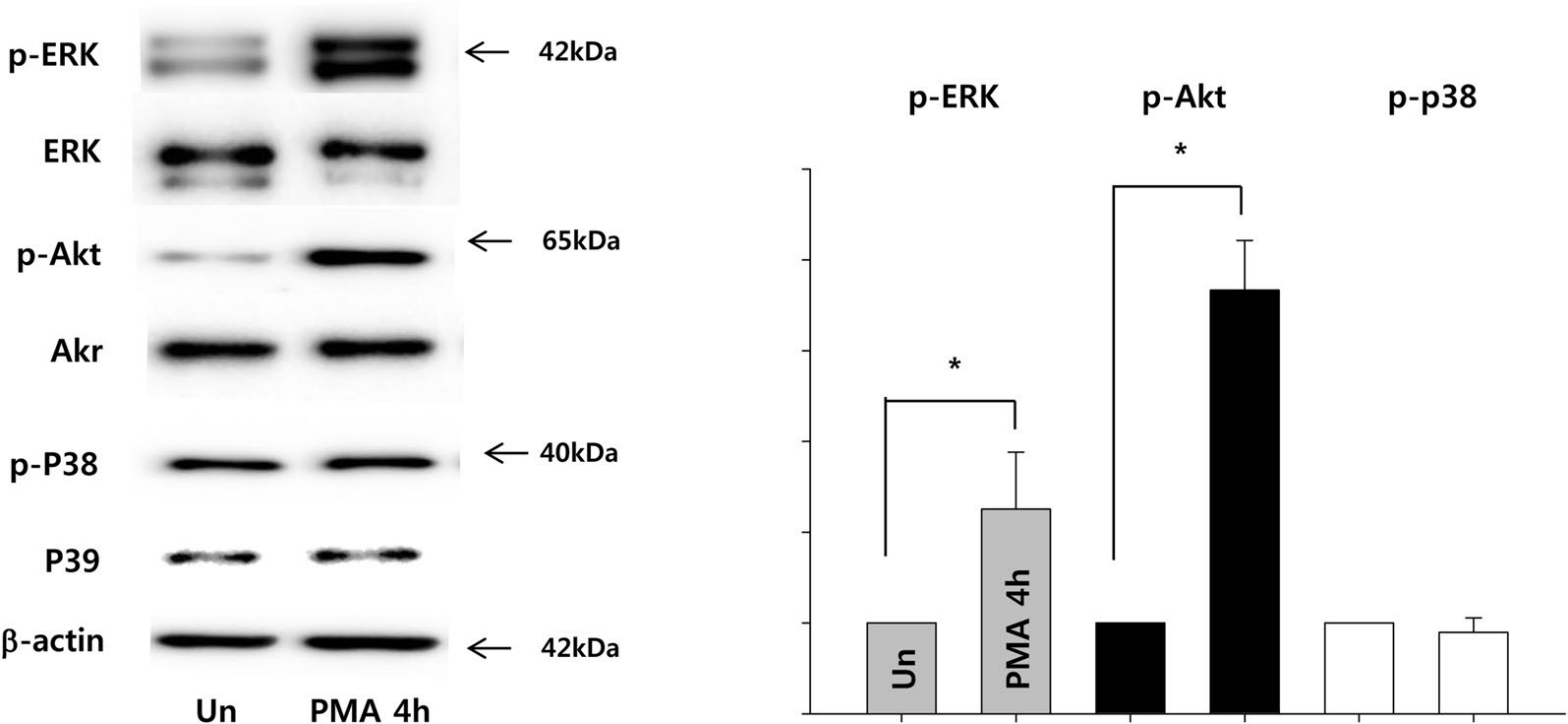
PMA activates ERK and Akt signaling in ADCS. ADSCs were treated with PMA (100 nM) for 4 h. The expressions of phosphorylated ERK (p-ERK), Akt (p-Akt) and p38 (p-p38) were normalized to total ERK, Akt, and p38, respectively. The quantitative data were expressed as the mean ± S.E.M of at least 3 independent experiments. *p < 0.05 compared to untreated control

Examination of anoikis resistant extracellular signal-regulated kinase (ERK) and Akt activation using Western blot indicated that the expressions of phosphorylated ERK and Akt significantly increased by 4 h of PMA treatment, while the expressions of phosphorylated p38 remained unaffected (Fig. 7).

## Discussion

One of the major problems of stem cell-based therapy is the low post-transplantation survival rate due to insufficient tissue integration called anoikis [31]. Therefore, preventing anoikis or enhancing initial cell adhesion may increase the therapeutic efficacy of stem cells for treating number of diseases including OA [8]. In this study, the effect of PMA on the ADSC adhesion to culture substrate and to a layer of chondrocyte was examined. Previous reports have demonstrated that PMA exerted cytotoxic effect on certain types of cells such as pancreatic cancer cells [32]. Therefore, we first verified whether there was any cytotoxic effect of PMA on ADSCs. As shown in the Fig. 1, PMA up to 100 nM did not cause any significant cytotoxic effect, and we moved on to further experiments.

PMA is a non-physiological activator of PKC [33]. Among different isozymes of PKC, which are comprised of conventional (PKCα, PKCβI, PKCβII and PKCγ), novel (PKCδ, PKCε, PKCη and PKCθ), and atypical (PKCζ and PKCι) isozymes, PMA activates conventional and novel PKCs by mimicking diacylglycerol (DAG) [34]. Regarding integrin-mediated cell adhesion, the importance of PKC activation and subsequent FAK phosphorylation and focal adhesion formation is well known [35]. Our data demonstrated that activation of PKC using increased ADSC adhesion and subsequent spreading in vitro (Figs. 2 and 3). However, we did not examine which type of isoenzymes were actually activated by PMA treatment so that it remains one of the limitations of this study, especially in elucidating the underlying mechanisms of the PMA-mediated ADSC adhesion enhancement. For future studies, using PKC inhibitors specific for each isoenzyme will be helpful to identifying the exact mechanism.

On the other hand, although PMA induced the phosphorylation of FAK required for cellular migration [36], it failed to enhance the migration of ADSCs (Fig. 4). This observation is somewhat contradictory to previous studies reported that PMA promoted cellular migration [37, 38]. Although we do not have data to back up at this point, one of the feasible explanations is that the use of 10% FBS supplemented DMEM for the experiment masked any possible effect of PMA on ADSC migration. In other words, if 0.1% FBS supplemented or serum free DMEM was used instead, the effect to PMA might have been detected. Another possibility is that we picked a wrong time point (4 h) to observe the early effect of PMA on ADSC migration. Since all the groups showed near-complete wound closing at 24 h, if there was any positive effect of PMA on cellular migration, such effect should have been observable between 4 and 24 h in our in vitro system. Therefore, for further studies, it is required to include 0.1% serum supplemented group and increase the number of observations for better understanding of the effect of PMA on ADSCs.

The important integrin-mediated signaling molecules such as FAK and integrin linked kinase (ILK) have been associated with cell survival, and these signaling molecules are also linked to PKC activity [39]. According to the data, PMA increased the transcription of key cellular adhesion molecules including FAK and talin after 4 h of treatment. Since both FAK and talin are critical components of integrin-containing adhesion complex that mediate cell–matrix interaction during cell spreading [40], PMA-induced increase of FAK and talin maybe the major factors mediated PMA-induced adhesion and spreading of ADSCs observed in the present study.

Furthermore, the protein level, the same concentration of PMA increased the protein expression of other integrin binding adhesion molecules, such as paxillin and vinculin [41]. Within the extracellular matrix, integrin associates with paxillin and vinculin, and it crosslinks actomyosin stress fibers which attached to focal contacts [42]. Subsequently, integrin clustering leads to association of the protein tyrosine kinase Src and the adaptor protein p130C to facilitate cellular adhesion [43]. Therefore, we speculate that PMA mediated activation of PKC, in turn, increased the expression of important components of focal adhesion complex to facilitate the adhesion of ADSCs.

Additionally, PMA treatment activated both ERK and Akt that are known to facilitate resistance to anoikis [44] in the present study. ERK is one of the major signaling of the mitogen-activated protein kinase (MAPK) signaling, and MAPK-dependent protection against apoptotic cell death has been well reported. For example, Bim is one of the factors that regulates anoikis [45] and the activation of MAPK suppresses Bim both at posttranslational [46] and transcriptional levels [47]. Furthermore, activation of MAPK upregulates the pro-survival members of the Bcl-2 family, such as Bcl-2, Bcl-XL and Mcl-1 [48]. Akt activation also promotes cell survival by preventing the release of cytochrome c from mitochondria [49] and inhibits the caspase cascade by phosphorylating the procaspase-9 [50]. Additionally PMA treatment did not affect p38 signaling, which is known to induce Anoikis [51]. Therefore, PMA-induced activation of survival signaling such as ERK and Akt without affecting p38 could be beneficial to the survival of transplanted ADSCs.

Current articular cartilage repair procedures often involve invasive processes such as microfracture surgery where damaged cartilage is drilled or punched to expose the underlying bone [52], and such procedure itself is discomforting and necessarily requires long and demanding rehabilitation [53]. As a non-invasive alternative approach, we envisioned that utilizing the PMA treated ADSCs for treating OA without invasive surgery, by directly injecting adhesion enhanced stem cells into the joint capsule without any major surgical procedure. Since the injected cells will be suspended in synovial fluid which is directly in contact with chondral heads (possibly damaged collagen fibers with exposed chondrocytes in OA patients), examining whether PMA treatment can enhance the interaction between ADSCs and chondrocytes should be the first step if the aforementioned non-invasive alternative approach were to be established.

Another important consideration to be taken is the nature of chemicals for enhancing the initial adhesion of ADSCs due to a possible carcinogenic effect. For example, PMA that used in the present study is known as a tumor promoter [54]. According to a “two-stage hypothesis” of carcinogenesis, there are two types of cancer-promoting agents; tumor initiators and tumor promoters. Tumor initiators are literally mutagens, whereas tumor promoters simply increase the number of cells carrying genetic defects caused by tumor initiators [55]. Therefore, the probability that PMA itself, as a tumor promoter, causes progression to malignancy seems to be very low [56]. Nevertheless, another study demonstrated that PMA indirectly induced sister chromatid exchanges by forming intermediate active oxygen species [57]. These contradicting reports suggest that extensive search of alternative chemicals that has no carcinogenic effect may be necessary to guarantee the clinical safety of the non-invasive approach we proposed. For the time being, as a proof of concept study, we examined whether PMA pretreated ADSCs had better interaction with chondrocytes.

Although the bound cells formed focal clumps and were relatively low in numbers, PMA treatment increased the number of bound ADSCs compared to untreated ADSCs (Fig. 5). The possible explanation for the modest number of attached cells to chondrocyte is the low seeding density. Since the experimental protocol was something we tried first without previous reference, the seeding density might have not been optimized. For example, for the experiments shown in Fig. 3, the cells were seeded in a chamber slide at a density of 1 × 10^3^/well, and that was reasonable to take pictures at lower magnification. However, for the experiments shown in Fig. 5, the cells were seeded at a density of 2 × 10^3^/well (of a 6 well plate) and the images were taken at higher magnification. The reason we did not used excessive amount of cells for seeding was that we expected most of the PMA pretreated cells would attach which was proven to be wrong. Therefore, the low seeding density might be one of the probable reasons of why the cells attached in modest numbers. Another possible reason may be the incubation time. Again, for the experiments shown in Fig. 3, the cells were allowed to attach for 6 h after the seeding while the cells were allowed for 4 h in experiment shown in Fig. 5. Therefore, for future experiments, higher seeding density and longer incubation time should be tried.

Nevertheless, Fig. 5 indicated that PMA may enhance the binding of ADSCs to the damaged chondral head for regeneration. In fact, according to our unpublished in vivo data, PMA-primed ADSC injection to the collagenase-treated articular cartilage of rats, which simulate wear and tear osteoarthritis in vivo [58], prevented further collagenase-induced degradation of the articular cartilage and maintained thicker cartilage layer compared to untreated OA controls. Nevertheless, we so far observed only the final improvement (data not shown) and not yet were able to find proper time point and experimental methods that can clearly demonstrate the PMA-induced early tissue integration of transplanted ADSCs compared to untreated ADSCs in vivo. Since our in vivo approach involves injecting cells into synovial fluid of knee joint rather than directly inject into cartilage tissues. Therefore, the injected cells have to superficially attach to the cartilage surface and then gradually be integrated. Because of this, it is always possible that loosely attached, or superficially attached cells, may be lost during sample preparation. To overcome such limitation of our current in vivo approach we used, we are trying to establish an ex vivo method of culturing explanted animal cartilage tissue in Matrigel. Once established, such method can be used to verify the effect of PMA pretreated ADSCs in treating OA.

## Conclusions

In summary, this paper describes small molecule-mediated enhancement of cell–cell interaction between ADSCs and chondrocytes. PMA significantly enhance the adhesion and spreading of ADSCs, as well as binding to chondrocytes in vitro, suggesting it may increase the possibility of ADSCs attach to damaged articular cartilage in sufficient number for regeneration in vivo. The result of this study indicates that priming ADSCs with PMA can be used to develop a non-invasive therapeutic approach for treating OA, minimizing unnecessary discomfort and pain to patients.

## Abbreviations

ADSC: adipose-derived stem cell
OA: osteoarthritis
PMA: phorbol 12-myristate 13-acetate
MSC: mesenchymal stem cell
FAK: focal adhesion kinase
ERK: extracellular signal-regulated kinase
MAPK: mitogen-activated protein kinase
